# Bilateral Ultrathin Descemet’s Stripping Automated Endothelial Keratoplasty vs. Bilateral Penetrating Keratoplasty in Fuchs’ Dystrophy: Corneal Higher-Order Aberrations, Contrast Sensitivity and Quality of Life

**DOI:** 10.3390/medicina57020133

**Published:** 2021-02-03

**Authors:** Massimo Castellucci, Costanza Novara, Alessandra Casuccio, Giovannni Cillino, Carla Giordano, Valentina Failla, Vincenza Bonfiglio, Maria Vadalà, Salvatore Cillino

**Affiliations:** 1 Department of Biomedicine, Neurosciences and Advanced Diagnostic, Ophthalmology Section, University of Palermo, 90127 Palermo, Italy; massimo.castellucci@gmail.com (M.C.); costanza.novara88@gmail.com (C.N.); giovannicillino@libero.it (G.C.); vale.failla@hotmail.it (V.F.); enzabonfiglio@gmail.com (V.B.); maria.vadala@unipa.it (M.V.); 2 Department of Health Promotion, Mother Child Care, Internal Medicine and of Excellence, University of Palermo, 90127 Palermo, Italy; alessandra.casuccio@unipa.it; 3 Department of Health Promotion, Mother and Child Care, Internal Medicine and Medical Specialties (ProMISE), Section of Endocrinology, Diabetology and Metabolism, University of Palermo, 90127 Palermo, Italy; carla.giordano@unipa.it

**Keywords:** bilateral ultrathin DSAEK, bilateral penetrating keratoplasty, visual acuity, contrast sensitivity, higher-order aberrations, quality of life

## Abstract

*Background and Objectives:* The objective of this paper is to compare the visual outcomes and quality of life (QoL) after bilateral ultrathin Descemet’s stripping automated endothelial keratoplasty (UT-DSAEK) with bilateral penetrating keratoplasty (PK) for Fuchs’ endothelial dystrophy (FED). *Materials and Methods:* Retrospective comparative cohort study, including 11 patients with FED who underwent bilateral PK and 13 patients with FED who underwent bilateral UT-DSAEK. All patients were already pseudophakic or had undergone a combined cataract procedure. The main outcomes were corrected distance visual acuity (CDVA) corneal higher-order aberrations (HOAs), contrast sensitivity (CS) and quality of life (QoL). *Results:* The mean follow-up after the second eye surgery was 32.5 ± 10.2 months in PK and 19.6 ± 8.6 months in UT-DSAEK patients. The CDVA in the UT-DSAEK group was significantly better than in the PK one (0.18 ± 0.07 vs. 0.35 ± 0.16 logMAR, *p* < 0.0001). The mean anterior corneal total HOAs of the central 5 mm were significantly lower in UT-DSAEK eyes than in PK eyes (0.438 ± 0.078 µ and 1.282 ± 0.330 µ respectively, *p* < 0.0001), whilst the mean posterior total HOAs did not differ between groups (0.196 ± 0.056 µ and 0.231 ± 0.089 µ, respectively, *p* = 0.253). The CS was lower at 0.75 and 1.5 cycles/degree in P the K group when compared to the DSAEK one (*p* = 0.008 and 0.005, respectively). The QoL scores by the NEI RQL-42 test exhibited better values in DSAEK patients in 9 out of 13 scales. *Conclusion:* Our study confirms that UT-DSAEK provides a better visual function in terms of CDVA and CS, together with lower HOAs, when compared to PK. Hence, the vision-related QoL, binocularly evaluated by the NEI RQL-42 items, indicates a higher satisfaction in UT-DSAEK eyes.

## 1. Introduction

For almost 15 years, Descemet’s stripping automated endothelial keratoplasty (DSAEK), and its subsequent evolution Descemet membrane endothelial keratoplasty (DMEK), have progressively replaced penetrating keratoplasty (PK) as the gold standard treatment in most cases of Fuchs’ endothelial dystrophy (FED) and post-surgical bullous keratopathy (BK) surgery [1,2]. This, besides the obvious advantage of the minimal invasiveness of the two lamellar procedures, is due to other typical disadvantages of PK, such as prolonged visual rehabilitation, high and often irregular astigmatism, unpredictable corneal power, suture-related complications, decreased postoperative wound strength, higher graft rejection rate, and anisometropia [3,4,5,6]. Therefore, there is general agreement that DSAEK and DMEK allow for better visual acuity, contrast acuity, avoidance of surgery-induced astigmatism and high order aberrations (HOAs) when compared to PK technique [4,5,7,8]. Even if some studies and meta-analyses report better visual acuity results, patients satisfaction and graft survival with DMEK compared with DSAEK, due to transplantation of isolated endothelium Descemet membrane layer without adherent corneal donor stroma in the former, the technical challenges related to DMEK ensure that, in 2018, DSAEK accounted for 65% of all endothelial keratoplasty surgeries [3,6,9,10,11,12,13]. Different studies have compared monocular long-term visual outcomes in DSAEK vs. PK in different groups, or DSAEK with PK in the fellow eye, or DSAEK with DMEK in the fellow eye, or even, by means of a questionnaire grading the symptoms and overall satisfaction with surgery on a scale of 1–6, patient satisfaction in contralateral comparison of DSAEK vs. PK or vs. DMEK [3,4,5]. Moreover, a multicenter prospective long-term study indicates that ultrathin DSAEK (UT-DSAEK) grafts, referring to grafts for the DSAEK technique not above 100 µ, result in better visual acuity compared with that of grafts over 145 µ. For some, UT-DSAEK grafts allow 5-year BSCVA, endothelial cell density, and survival rates comparable with DMEK, even if with a higher immunologic rejection rate [14,15,16].

This retrospective study aims to compare corrected distance visual acuity (CDVA), HOAs, contrast sensitivity (CS) and quality of life (QoL), through the National Eye Institute Refractive Error Quality of Life Instrument-42 (NEI RQL-42), after bilateral UT-DSAEK versus bilateral PK for FED or BK in pseudophakic patients.

To the best of our knowledge, no refractive error-related QoL evaluation through a validated questionnaire, such as the NEI RQL-42, has previously been performed in these kinds of patients after a bilateral procedure.

## 2. Materials and Methods

This retrospective comparative cohort study was approved by the Ethics Committee of the University of Palermo and followed the tenets of the Declaration of Helsinki (R.B. approval No. 4/2015, date of approval 15 April 2015). We reviewed the medical charts of patients with FED or BK who had undergone bilateral PK or bilateral UT-DSAEK from January 2008 to December 2017 at the Department of Ophthalmology of the University of Palermo, Italy. Data collected included preoperative corrected distance visual acuity (CDVA) and FED stage, including BK [17], and on the last postoperative follow-up visit, CDVA, manifest refraction, corneal HOAs of the central 5 mm zone, spatial frequency contrast sensitivity, and degree of satisfaction assessed by the NEI RQL-42 test. Only patients with digitally stored reliable corneal tomographic maps, and in which the above tests had been performed and recorded months after suture removal, were included.

Patients affected with diseases that would limit visual outcomes, such as age-related macular degeneration, advanced glaucoma, anterior segment or fundus abnormalities, were excluded. Rejections, graft failures, and complications other than rebubbling in UT-DSAEK cases were excluded from the analysis. Patients with secondary cataract had undergone prompt YAG Laser capsulotomy. 

Until 2012, PKs had mainly been performed, whilst thereafter DSAEK was almost the exclusive procedure. The interval between the 1st and 2nd eye surgery ranged from 8 to 24 months in PK patients, and from 6 to 18 months in UT-DSAEK cases. If the eye was phakic, a triple procedure, i.e., cataract extraction, intraocular lens implantation, and corneal graft, was performed in both groups. The last postoperative follow-up visits we considered in patients who underwent PK had been performed not less than 6 months after the 2nd eye suture removal, while in UT-DSAEK eyes, the last visit had taken place at least 10 months after the 2nd surgery, and at least 3 months after suture removal, ensuring refractive stabilization. 

### 2.1. Surgical Procedures

All surgeries had been performed by one skilled surgeon (SC). The PK procedure involved trephining, the recipient cornea with a Hessburg-Barron vacuum trephine, centered on the geometric center, and fashioning a 0.25 mm oversized donor cornea punched from the endothelial side with a Barron donor punch (Jedmed Instrument Co, St Louis, MO, USA). Excision of the recipient corneal button was completed with curved corneal scissors. The donor cornea was secured with 10-0 nylon (Alcon Laboratories, Inc. Fort Worth, TX, USA) by double continuous 16-bite non-torque running sutures [18,19]. In patients with crystalline lens, cataract extraction and IOL placement were performed immediately before keratoplasty, by phacoemulsification, or during the keratoplasty procedure using extracapsular extraction. 

The UT-DSAEK surgeries were performed using pull-through technique. Precut UT-DSAEK discs about 8 to 8.5 mm in diameter were provided by the Veneto Eye Bank Foundation (FBVO, Mestre, Italy) using an automated lamellar therapeutic keratoplasty system. In detail, graft dissection had been performed with a Moria microkeratome (Evolution-3), equipped with either a 300 or 350 mm-deep blade depending on the thickness of the cornea, passed over the tissue with a targeted posterior lamella thickness of 100 ± 20 µm for UT-DSAEK. Donor and lamellar central corneal thickness were measured at the cornea bank using an anterior segment Fourier-domain optical coherence tomographer (OCT Casia SS-1000; Tomey GmbH, Tomey Corporation, Erlangen, Germany). The storage medium consisted of MEM Earle with 25 mM of Hepes, 26 mM of sodium bicarbonate, 1 mM of pyruvate, 2 mM of glutamine, and 2% (vol/vol) of newborn calf serum, 0.25 mg/mL, amphotericin B, 60 mg/mL penicillin G, and 100 mg/mL of streptomycin and 6% (wt/vol) of dextran-T500. When necessary, the host central corneal epithelium was removed with a blunt spatula to improve visualization. Thereafter, the corneal surface was marked with a corneal DSEK marker (Janach^®^ srl, Como, Milan, Italy) stained with gentian violet dye. Through a temporal near-clear 1.5 mm tunnel, a scoring reverse hook (Janach^®^ srl, Como, Milan, Italy) was used to incise the host endothelium and Descemet’s membrane along the circumference corresponding to the epithelial mark. After Descemet’s membrane with host endothelium stripping and insertion of an inferiorly placed 25-gauge anterior chamber (AC) maintainer, the peripheral posterior stroma was scraped to improve lenticule adherence. Contrary to the temporal tunnel, a nasal 4.2 mm tunnel was fashioned. The tunnels were used to pull in the donor cornea lenticle through the nasal one using a “closed chamber” Macaluso inserter [20], and a 23-gauge endothelial lenticle forceps (Janach^®^ srl, Como, Milan, Italy). In phakic eyes, cataract phacoemulsification with IOL implantation was performed through temporal tunnel before corneal epithelium marking. Wounds were closed with 10-0 nylon single bite sutures, and air tamponade was achieved with a large bubble in the anterior chamber for 10 min. Subsequently, air was partially removed, leaving a smaller air bubble in the AC, and a bandage contact lens was placed. 

In phakic eyes in both groups, one-piece aspheric hydrophobic intraocular lenses (IOLs), Tecnis ZCB00 (AMO, Johnson-Johnson Vision, Dublin, Ireland) or Acrysof IQ (Alcon Italia Spa, Milan, Italy), had been inserted. 

All PK and UT-DSAEK patients received a postoperative steroid regimen including topical 0.1% dexamethasone sodium phosphate and tobramycin antibiotic (Tobradex; Alcon Italia S.P.A., Milan, Italy), every 3 h for 7 days, then topical 0.15% dexamethasone sodium phosphate 4 times daily for 2 months, which was tapered by 1 drop every 3 months to 1 drop daily by 1 year, and thereafter 1 drop each other day was continued indefinitely, unless steroid-induced glaucoma had been diagnosed. In PK cases, the suture removal had been performed 12 to 16 months postoperatively. 

### 2.2. Outcome Measures

Clinical data recording had been performed before keratoplasty procedures, and after 24 h, 1 week, 1, 3, 6, 12 months, and till the 3rd to 4th year postoperatively. Only preoperative data and data from the last suitable clinical examination were analyzed due to incomplete records in some cases at various follow-up time points. 

As above, outcomes were the long-term differences between PK and UT-DSAEK in terms of manifest refraction, CDVA, corneal HOAs of the central 5 mm zone, spatial frequency contrast sensitivity, and degree of satisfaction assessed by the NEI RQL-42 test. 

Monocular CDVA was measured in logMAR notation at 100% contrast using Early Treatment of Diabetic Retinopathy Study charts under photopic conditions (CC-100XP LCD System for Chart display, Topcon Europe BV, Milano, Italy) at 4 m. 

The measurement of corneal HOAs from the anterior and posterior corneal surface was performed with a Scheimpflug-camera system (Sirius CSO, Florence, Italy). The Sirius imaging apparatus includes a Placido disk and a noncontact 360-degree rotating Scheimpflug camera, by which the anterior and posterior corneal heights and pachymetric data are obtained. The camera scans the anterior and the posterior surface of the cornea in seconds and obtains three-dimensional maps, relying on 21,632 real measuring points in the front surface, and 16,000 measuring points in the rear surface. A resident software (Phoenix Software-Suite, CSO, Florence, Italy) elaborates the anterior and posterior height data to Zernike polynomials, extracts the components of the best-fit sphere and multiplies the residual components by the difference in the refractive indices at the anterior and posterior surfaces. The resulting corneal HOAs values in µm we analyzed include the root mean square (RMS) of the total anterior and posterior HOAs, defined as the RMS of the magnitudes of the 3rd-to 6th-order aberrations, and the RMS of the magnitude of the anterior and posterior coma, trefoil, and spherical aberration (SA) (Z^1^_3_/Z^−^^1^_3_, Z^3^_3_/Z^−^^3^_3_, and Z^0^_4_, respectively, by Zernicke terms). All HOAs were calculated for 5 mm pupils to simulate mesopic conditions. 

Contrast sensitivity (CS) was determined monocularly, using sine-wave gratings through a computerized spatial frequency contrast sensitivity test (CC-100XP LCD System Chart System; Topcon Europe BV, Milano, Italy), which is customized by setting the amount of contrast levels, at six spatial frequencies, from 0.75 to 18 cycles/degree, in photopic condition at 4 m. The resulting patient’s contrast sensitivity curve is displayed in a graph and also shows the normal population range as a grey area. 

Patient satisfaction was assessed by the NEI RQL-42 test to evaluate vision-related QoL. All the 42 items in the NEI-RQL are grouped into 13 scales covering specific aspects of QoL. Each of the 13 subsets is composed of 1 to 7 items, the scores of which are averaged to yield the final score for that subset. Each scale has a score from 0 to 100. A higher score on the NEI RQL-42 scales indicates a higher self-reported QoL [21,22].

### 2.3. Statistical Analysis

Statistical analysis of quantitative data, including descriptive statistics, was performed for all items. All continuous data are expressed as a mean ± standard deviation of the mean. All visual acuity results are presented in a logarithm of the minimal angle of resolution units. Comparisons between categorical variables were conducted using the Fisher exact or chi-square test, as appropriate. For continuous measures, Student t test or for nonparametric Mann-Whitney U test were used.

The paired Wilcoxon signed-rank test was used for intragroup analysis. Preoperative CDVA differed significantly between the groups.

We performed analysis of covariance (ANCOVA), used to adjust for pre-existing differences in nonequivalent groups that cannot be made equal through random assignment.

Data were analyzed by IBM SPSS Software version 22.0 (SPSS, Inc., Chicago, IL, USA). All *p* values were two-sided, and *p* values less than 0.05 were considered statistically significant.

## 3. Results

The records of 24 patients (48 eyes) were suitable for analysis. Eleven patients had undergone bilateral PK and 13 patients bilateral UT-DSAEK (Table 1). No statistically significant differences between groups were found regarding the mean age (range 62–77 years) and male/female relationship. The preoperative FED stage was significantly worse in PK eyes (*p* < 0.0001). In more detail, even if not always clearly described in our preoperative charts, stromal or subepithelial fibrosis was presumably present in 12 out of 22 eyes (54%) in the PK group, and in 6 out of 26 eyes (23%) in the UT-DSAEK one. Preoperative CDVA was significantly better in the UT-DSAEK group (0.68 ± 0.12 logMAR) than in the PK group (1.08 ± 0.45 logMAR) group (*p* < 0.0001). The percentage of triple combined cataract procedures was higher in the UT-DSAEK group (*p* < 0.05). The mean graft thickness in UT-DSAEK eyes, as measured by recorded data from the Eye Bank, was below 100 µ (range 61–137 µ), with a graft ≤100 µ in 60% and <130 µ in 95% of eyes. In four eyes (15%) of the UT-DSAEK group, a successful rebubbling with air was performed during the first few postoperative days due to graft detachment. Mean follow-up after the second eye surgery was above two and a half years in the PK group and above one and a half year in UT-DSAEK group.

Postoperative CDVA improved in both groups at the last follow-up visit (Table 2; *p* < 0.0001) and was better in the UT-DSAEK eyes with respect to PK ones (*p* < 0.0001). PK cases exhibited significantly higher negative manifest sphere and cylinder than UT-DSAEK (*p* < 0.001 and < 0.05 respectively). Correcting for preoperative CDVA by ANCOVA analysis confirmed that UT-DSAEK patients had significantly better postoperative CDVA (*p* = 0.015).

The mean RMS of the anterior and posterior corneal HOAs of the central 5 mm zone in the PK eyes and UT-DSAEK eyes are shown in Table 2. Both anterior total HOAs and the single anterior Zernike coefficients we considered were significantly higher in the PK group than in the UT-DSAEK group (*p* < 0.0001). There was no significant difference regarding total HOAs nor any of the HOAs Zernike terms of the posterior corneal surface.

**Figure 1:**
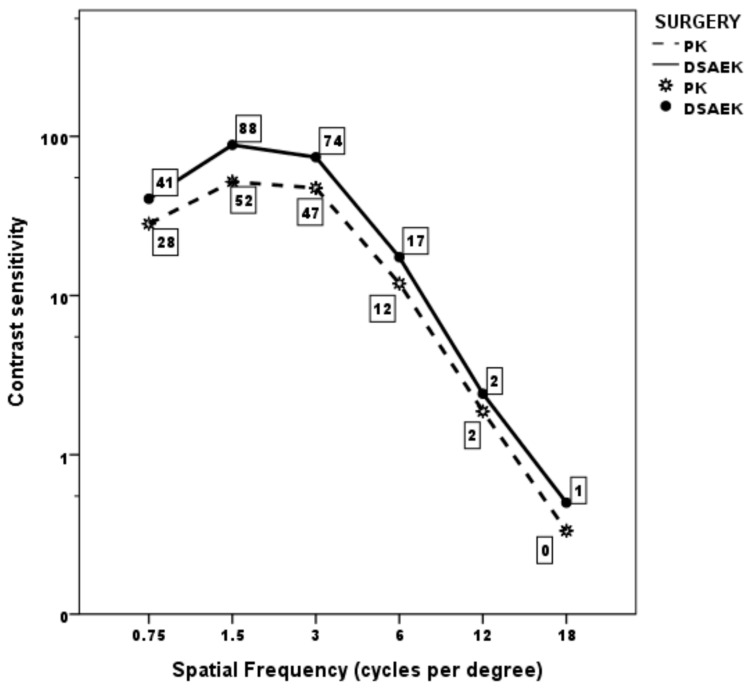
Mean monocular contrast sensitivity curve in the two groups at last postoperative follow-up visit.

**Figure 2:**
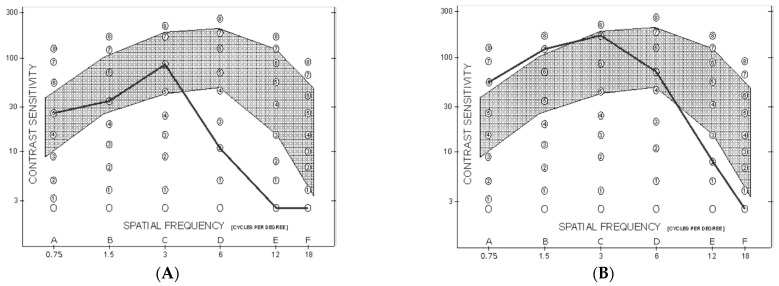
Monocular spatial-frequency contrast sensitivity curve at last postoperative follow-up visit. The grey interval indicates the normal range. (**A**): Eye that underwent PK, 6 months after suture removal. (**B**): Eye that underwent UT-DSAEK 10 months after surgery.

Mean monocular CS curve was within normal population limits from 0.75 to 3 cycles/degree in both the PK and UT-DSAEK group, albeit with a significant score difference favoring the latter at 0.75 and 1.5 cycles/degree (*p* = 0.008 and 0.005, respectively; Table 3; Figure 1). Thereafter, it began to fall below the limits at higher frequencies, without difference, in both groups. Examples of the CS curve in two cases, respectively, that underwent PK and UT-DSAEK are shown in Figure 2. 

**Figure 3:**
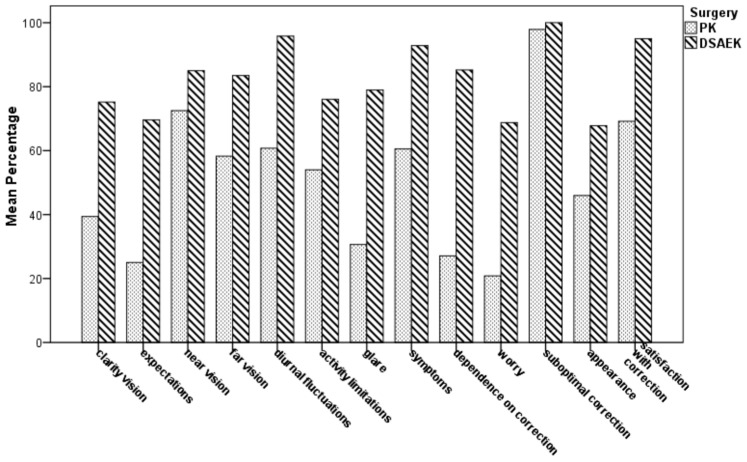
Comparison of mean scale scores of the National Eye Institute Refractive Error Quality of Life Instrument-42 (NEI RQL-42) for vision-related Quality of Life (QoL) between PK and UTDSAEK patients at the last postoperative follow-up visit.

Table 4 and Figure 3 show the postoperative vision-related QoL scale scores by the NEI RQL-42 test in both groups. In 9 out of 13 subset scales, the mean score was significantly higher in the UT-DSAEK group than in the PK group (*p* ranging from 0.013 to <0.0001). No differences were found in “near vision”, “activity limitations”, “suboptimal correction”, and “appearance” subscales.

## 4. Discussion

As pointed out in the Introduction, DSAEK, whose advantages over PK have been proven for at least ten years [3,4,5], has evolved bi-directionally towards techniques employing much thinner grafts such as DMEK or UT-DSAEK. This trend depends on the evidence of improved visual outcomes with thinner DSAEK tissue [23,24,25]. As already described, prospective and retrospective studies indicate that UT-DSAEK allows for faster and better CDVA recovery with respect to DSAEK, and CDVA, endothelial cell density, and survival rates comparable to DMEK [14,15,16]. Some randomized, controlled clinical trials (RCTs) and retrospective studies indicate that DMEK, whose graft thickness is 10–15 µ, allows for superior visual acuity and lower posterior corneal HOA, compared to UT-DSAEK until 12 to 24 months postop. However, though literature data agree in indicating that DMEK, as said above, involves high surgical skill, and it is characterized by a steep learning curve, complex graft preparation and handling with a high risk of endothelial trauma, and frequent postoperative graft detachment requiring air-gas reinjection, referred to as rebubbling [6,10,12,13,26,27]. Therefore, many surgeons stay with DSAEK, or even better with its refinement UT-DSAEK, implying a reduction of graft average central thickness from 200 µ to 100 µ [14,15,16,23,24]. Recently, a further evolution of UT-DSAEK has been proposed, described as “nanothin DSAEK (NT-DSAEK)”, using grafts within 50 µ, which should provide visual outcomes and complications rates that are comparable to DMEK [28,29].

In contrast to what has been described so far, a recent hospital-based retrospective study on 150 eyes that underwent endothelial keratoplasty with various techniques and 32 months of average follow-up time, stated that DMEK and NT-DSAEK were better than DSAEK only relating to the rapidity of visual recovery. The final quality of vision, in terms of CDVA and HOAs, and rate of graft failure were not related to the graft thickness and regularity [30].

We have to remark that, as stated in the Introduction, penetrating keratoplasty is not performed anymore in FED or pseudophakic BK, with the exception of patients with massive stromal or subepithelial fibrosis, corneal scars or a complex anterior segment situation such as deep anterior chamber, large iris defects or artificial iris. Our PK cases group depends on both the advanced FED stage and on a time when DSAEK was not yet practiced at our facility, as stated in the Materials and Method Section. The worse preoperative FED stage and monocular CDVA in our PK eyes indicates that these were operated at a later stage of the disease. This finding could depend on the habit to delay PK procedures with respect to DSAEK ones, due to the potential intraoperative and postoperative complications of the former. The higher percentage of triple procedures in UT-DSAEK group with respect to the PK one, indirectly confirms that, in the former, more often the surgeon simultaneously dealt with both diseases due to the low complications risk, whilst in the latter, keratoplasty was delayed for as long as possible. 

As previously exposed, clinically significant complications were ruled out in the two groups of patients included in the study, except for the 15% graft detachment rate followed by rebubbling in UT-DSAEK cases. Our rebbubling rate is higher with respect to some studies, but in agreement with one multicenter RCT [12,15,16]. 

The higher CDVA described with all endothelial keratoplasty techniques with respect to PK in Fuchs’ dystrophy, and primarily due to higher sphero-cylindrical error with suture-related irregular astigmatism with the latter [4,5,6,8,9,15], is confirmed in our study, where UT-DSAEK eyes achieved better CDVA when compared to PK ones, with lower degrees of sphero-cylindrical correction. This result, besides the inherent refractive disadvantages of the PK technique, could also be ascribed to the more advanced stage of disease in the PK group, which implicitly indicates a greater number of BK cases, with greater alteration of the peripheral recipient bed and increase of suture irregularities. Another factor could be the extensive corneal nerve damage with associated effects on tear film quality in PKs [4,25]. ANCOVA correction for the significantly worse CDVA in PK patients confirms, in any case, significantly better endpoint vision in UT-DSAEK eyes, regardless of the preoperative condition. One long-term prospective interventional case series, performing a sub analysis of FED versus BK in DSAEK and PK cases [4], found that DSAEK led to better visual acuity outcomes than PK, particularly in FED. In BK, the superiority of DSAEK over PK was not evident, probably due to irreversible stromal scarring. In these cases, PK can still be a viable indication. 

Another aspect to be highlighted is the small postoperative hyperopia in the UT-DSAEK group. It has been demonstrated that the difference in thickness between the center and periphery of the DSAEK grafts modifies the posterior corneal curvature, causing a hyperopic shift that decreases with time until often-negligible values. This decrement is due to deswelling of the periphery of the donor button with scarring of the peripheral stroma [31]. The relatively low value (+0.37 ± 0.80 diopters) in our patients can have been further reduced thanks to the small UT-DSAEK thickness. 

In our study, the anterior total HOAs and the three different anterior Zernike coefficients (coma, trefoil and SA) were significantly higher in the PK group than in the UT-DSAEK group. It has been reported that the above HOAs coefficients account for nearly 80% of the HOAs in DSAEK subjects [32]. The higher amount of HOAs in PK, for a long time confirmed by several studies [5,7,8,9], is ascribed to mismatched thicknesses of the donor-host junction, different radius of curvature between donor lenticule and host cornea, suture irregularities. All these result in the abovementioned high sphero-cylindrical error and irregular astigmatism, which are in turn responsible for the anterior HOAs. Besides the higher value of anterior corneal coma and SA in PK vs. DSAEK eyes, previous studies have confirmed the equivalence of the same parameters with normal eyes in the latter [7,9]. This seems rather obvious since no endothelial keratoplasty technique modifies the corneal anterior curvature, the only factor for increased HOAs with respect to healthy eyes consisting of subepithelial fibrosis in more advanced FED stages [16]. 

On the contrary, the posterior total HOAs and the three different posterior Zernike coefficients (coma, trefoil and SA) did not differ in the two groups. This finding is in contrast with some studies [7,33] where both higher posterior total HOAs and higher specific Zernike coefficients in DSAEK cases in comparison to PK were reported. These authors pointed out that total corneal aberration and optical function are mainly determined by the anterior surface, although there is some compensation by the posterior surface after PK, in which the two surfaces are maintained parallel and are similar to each other. In DSAEK, where the posterior surface is replaced, no compensation for anterior HOAs is foreseeable, so that in some patients, an increase up to 20% of the total HOAs is due to the unmatched anteroposterior surfaces [33], since the difference between the corneal anterior surface refractive index and air is tenfold higher than that between the endothelial surface and aqueous, the effect of posterior surface on total corneal HOAs is regarded as much lower [34]. A significant correlation of CDVA with DSAEK graft thickness has been reported, with thinner grafts associated with greater visual gain, and thicker grafts associated with greater asymmetry of the posterior surface, which in turn was associated with more posterior HOAs [32,35]. Contrary to what has been discussed so far, other studies indicate that DSAEK and PK exhibit similar posterior corneal HOAs increase, even years after surgery, whereas DMEK cases, characterized by simple Descemet’s membrane with endothelium apposition, display only slight changes in posterior corneal HOAs with respect to control eyes [8,9]. In summary, posterior HOAs appear to be related to the anterior ones in PK and to graft thickness in DSAEK, so that, in the latter case, the minimal thickness of the DMEK variant allows for less pronounced and more physiologic HOAs. It seems likely therefore, based on what we have said so far, that discrepancies among studies could well be related to different graft thicknesses in different samples. 

Even if the UT-DSAEK technique could have generated fewer posterior HOAs in our cases, we found equivalence between groups. This could be related to various factors such as sample size, percentage of grafts above 100 µ, influence of dislocation with rebubbling procedures and graft imperfect centering. We did not analyze these aspects in detail due to the small dimension of these subgroups. 

Significant postoperative improvement in CS and better contrast acuity after DSAEK in comparison with PK have been previously described [5,36]. We point out that, in our cases, the aspheric pseudophakic condition can avoid variations on CS arising from the natural crystalline lens in elderly patients [3,5,37]. The monocular photopic CS, measured through intermediate distance recognition of sinusoidal gratings, was normal in both groups at low spatial frequencies, even if at the lowest frequencies it was higher in UT-DSAEK group with respect to the PK one. This finding could be related to the significantly lower anterior HOAs in the UT-DSAEK group than in the PK group. At higher frequencies, on the contrary, both groups fall below normal CS, with no differences, notwithstanding a better CDVA and lower HOAs in UT-DSAEK eyes. In one study where an adaptive optics system was used to correct ocular HOAs, PK eyes obtained better VA than that of DSAEK eyes, suggesting that the decreased VA in the DSAEK population could also be partially related to corneal haze [32]. According to these and other authors, this haze, responsible for light scatter, could be subepithelial and related to anterior stromal fibrosis in chronic corneal edema or may be due to donor–host stroma/stroma interactions at the interface [32,38]. The above considerations could explain the low CS even in our UT-DSAEK subjects at high frequencies, despite the lower level of HOAs, especially considering the incidence of subepithelial fibrosis described in the results. Under this respect, some authors found a better contrast ratio with Landolt ring in the DMEK operated eye than in the contralateral DSAEK operated eye. They attribute this finding to the thickness of the transplanted lamella and to the stroma–stroma interface [3].

The NEI RQL-42 test has been widely recognized as a valuable measurement of the refractive error-related QoL [22,39,40]. The higher mean scores in 9 out of 13 subset scales grouping the NEI RQL-42 items in UT-DSAEK eyes indicates a better vision-related QoL in these patients. High scores in relevant scales, such as clarity of vision, far vision, diurnal fluctuations, glare, dependence and satisfaction with correction, all indicate a high degree of satisfaction in the UT-DSAEK group. The lack of intergroup difference in four scales, i.e., near vision, activity limitations, suboptimal correction and appearance, could depend on factors such as pseudophakic presbyopic condition, significantly less activity limitation with both techniques, less strict relationship with refraction, or small sample size. Trousdale and coworkers analyzed the vision related QoL after three types of keratoplasty (penetrating keratoplasty (PK), deep lamellar endothelial keratoplasty (DLEK), and descemet stripping endothelial keratoplasty (DSEK)) in FED patients, using the 25-item National Eye Institute Visual Functioning Questionnaire (NEI VFQ-25), through a prospective, observational case series [41]. They conclude that QoL in patients with FED improves after keratoplasty, irrespective of the technique, with a faster improvement after DSEK than after PK. However, it should be pointed out that many of their patients had undergone monocular keratoplasty, or PK in one eye and DLEK or DSEK in the fellow eye, or bilateral DSEK, with an apparent mixture of the different populations. Moreover, the NEI VFQ-25 test is not designed to distinguish individuals with corrected refractive error from emmetropic individuals who have normal vision without correction, and it is best suited for macular degeneration or glaucoma-related loss of vision [40,42].

Limitations of the present study are the retrospective design, which implies that PK was performed in more advanced FED stages, with different pre-operative clinical groups, and the lack of analysis of subgroups with BK, due to small sample size, which could have implied worse results, especially in UT-DSAEK cases. Another limitation is lacking endothelial cell density data at the last follow-up visit in our groups. Moreover, data on postoperative subepithelial or posterior interface fibrosis in UT-DSAEK cases, and their influence on HOAs and CS, are lacking.

## 5. Conclusions

In conclusion, our study confirms that UT-DSAEK allows for better long-term CDVA with lower degrees of sphero-cylindrical correction when compared to PK. The latter is also affected by higher anterior HOAs, which could, in turn, be responsible for lower CS at low frequencies. A higher CS sensitivity in UT-DSAEK could not be confirmed at high spatial frequencies, perhaps due to some degree of anterior fibrosis in more advanced cases or posterior interface with thicker grafts. Finally, refractive error-related QoL evaluation through the NEI RQL-42 questionnaire indicates better vision-related QoL in patients who had bilaterally undergone UT-DSAEK, which is therefore confirmed as a first-choice surgical technique in FED. 

## Figures and Tables

**Table 1:** Patient baseline characteristics.

	PK	UT-DSAEK	*p*
No. of eyes	22	26	
Age	68.6 ± 4.8	67.6 ± 2.8	0.373
M/F	5/6	6/7	1.0
FED Stage	3.6 ± 0.5	2.3 ± 0.5	**<0.0001**
PREOP CDVA (logMAR)	1.08 ± 0.45	0.68 ± 0.12	**<0.0001**
Phakic (%)/pseudophakic	6(27%)/16	15(58%)/11	**0.044**
UT-DSAEK mean graft thickness (µ)	-----	99.25 ± 19.40	----
UT-DSAEK rebubbling eyes (%)	-----	4 (15%)	----
Follow-up after 2nd eye surgery (Mo)	32.5 ± 10.2	19.6 ± 8.6	**<0.0001**

PK = Penetrating keratoplasty; UT-DSAEK = Ultrathin Descemet’s stripping automated endothelial keratoplasty; FED = Fuchs’ endothelial dystrophy; CDVA = corrected distance visual acuity. Statistically significant values appear in bold.

**Table 2:** Visual acuity, manifest refraction, and root mean square of high order corneal aberrations in the central 5 mm zone in penetrating keratoplasty and ultrathin Descemet’s stripping automated endothelial keratoplasty groups at the last postoperative follow-up visit: the corresponding Zernike term are in parentheses.

	PK	UT-DSAEK	*p*
Eyes (Patients)	22 (11)	26 (13)	
CDVA *	0.35 ± 0.16	0.18 ± 0.07	**<0.0001**
Manifest Sphere ^§^	−0.96 ± 1.67	+0.37 ± 0.80	**0.0008**
Manifest Cylinder ^§^	−1.40 ± 2.50	−0.21 ± 1.38	**0.042**
Anterior: Total HOAs	1.282 ± 0.330	0.438 ± 0.078	**<0.0001**
Coma (Z_3_^1^)	0.473 ± 0.228	0.193 ± 0.090	**<0.0001**
Trefoil (Z_3_^3^)	0.392 ± 0.202	0.131 ± 0.066	**<0.0001**
SA (Z_4_^0^)	0.737 ± 0.261	0.301 ± 0.085	**<0.0001**
Posterior: Total HOAs	0.231 ± 0.089	0.196 ± 0.056	0.253
Coma (Z_3_^1^)	0.103 ± 0.043	0.091 ± 0.042	0.334
Trefoil (Z_3_^3^)	0.095 ± 0.038	0.092 ± 0.024	0.741
SA (Z_4_^0^)	0.090 ± 0.071	0.060 ± 0.036	0.065

PK = Penetrating keratoplasty; UT-DSAEK = Ultrathin Descemet’s stripping automated endothelial keratoplasty; CDVA = corrected distance visual acuity; * Postop vs preop *p* value < 0.0001, both in PK and UT-DSAEK. ^§^ Diopters. HOAs = corneal higher-order aberrations; Coma = coma aberration; Trefoil = Trefoil aberration; SA = Spherical aberration. Statistically significant values appear in bold.

**Table 3:** Mean contrast sensitivity score at different spatial frequencies in the PK group and UT-DSAEK group at the last postoperative follow-up visit.

Spatial Frequency (CPD)	PK	UT-DSAEK	*p*
0.75	27.68 ± 15.54	41.57 ± 18.83	**0.008**
1.5	52.50 ± 38.87	88.84 ± 46.09	**0.005**
3.0	48.95 ± 48.65	74.80 ± 52.85	0.086
6.0	12.50 ± 14.73	17.30 ± 17.10	0.307
12.0	2.04 ± 2.73	2.57 ± 2.64	0.498
18.0	0.36 ± 1.18	0.46 ± 1.30	0.783

PK = Penetrating keratoplasty; UT-DSAEK = Ultrathin Descemet’s stripping automated endothelial keratoplasty; CPD = Cycles per degree. Statistically significant values appear in bold.

**Table 4:** Vision-related Quality of Life Scores (Mean ± Standard Deviation) by National Eye Institute Refractive Error Quality of Life Instrument-42 Test in PK group and UT-DSAEK group at the last postoperative follow-up visit.

	PK (11 Patients)	UT-DSAEK (13 Patients)	*p*
Scale 1 Clarity of vision	38.83 ± 4.87	75.00 ± 24.011	**<0.0001**
Scale 2 Expectations	25.00 ± 27.39	69.23 ± 48.038	**0.013**
Scale 3 Near vision	72.35 ± 22.98	85.10 ± 9.7289	0.082
Scale 4 Far vision	57.53 ± 18.42	83.97 ± 12.501	**0.0004**
Scale 5 Diurnal fluctuations	59.85 ± 12.81	96.15 ± 7.3088	**<0.0001**
Scale 6 Activity limitations	54.92 ± 37.76	75.00 ± 34.985	0.190
Scale 7 Glare scale	30.68 ± 15.17	79.81 ± 20.116	**<0.0001**
Scale 8 Symptoms	59.74± 12.38	92.58 ± 5.147	**<0.0001**
Scale 9 Dependence on correction	26.92 ± 33.011	86.36 ± 14.92	**<0.0001**
Scale 10 Worry	20.45 ± 10.11	69.23 ± 48.038	**0.003**
Scale 11 Suboptimal correction	97.73 ± 7.54	100.00 ± 1.25	0.295
Scale 12 Appearance	45.45 ± 35.22	67.95 ± 19.792	0.061
Scale 13 Satisfaction with correction	69.09 ± 22.56	95.38 ± 8.7706	**0.0008**

PK = Penetrating keratoplasty; UT-DSAEK = Ultrathin Descemet’s stripping automated endothelial keratoplasty. Statistically significant values appear in bold.

## Data Availability

The data presented in this study are available on request from the corresponding author. The data are not publicly available due to their sensitive nature as ‘data concerning health’.
